# Transection of the glossopharyngeal nerve reduces energy and sugar intake but does not affect fat intake or meal patterns in rats offered a palatable cafeteria diet

**DOI:** 10.1016/j.physbeh.2026.115268

**Published:** 2026-02-12

**Authors:** Carolina R. Cawthon, A.Valentina Nisi, Ginger D. Blonde, Alan C. Spector

**Affiliations:** a Department of Psychology, 1107 W Call Street, Florida State University, Tallahassee, FL 32306, USA; b Department of Nutrition, 1215 W Cumberland Ave, 229 Jesse Harris Building, University of Tennessee-Knoxville, Knoxville, TN 37996, USA; c Program in Neuroscience, 1107 W Call Street, Florida State University, Tallahassee, FL 32306, USA

**Keywords:** Gustatory nerves, Glossopharyngeal nerve, Food choice, Meal patterns, Nerve transection

## Abstract

Taste information is transmitted to the brain via branches of cranial nerves VII (chorda tympani, CT, and greater superficial petrosal, GSP), IX (glossopharyngeal, GL), and X (superior laryngeal branch of the vagus) with each nerve innervating relatively segregated fields of taste buds on the tongue, palate, and laryngeal epithelium. Approximately 70% of taste buds reside on the tongue and are innervated by the CT or GL. Previously we found that rats offered a 5-choice cafeteria diet after combined transection of the CT and GL (2NX) ate more fat, less sugar, and larger, but fewer meals each day resulting in no net change in total daily energy intake and suggesting a role for the CT and/or GL in food choice, regulation of macronutrient intake, and satiation signaling, but the role of the individual nerves was unclear. To begin identifying the role of each nerve, this experiment aimed to interrogate the effects of transection of the GL (GLX) on food choice and meal patterns in rats offered a cafeteria diet after recovery from GLX or SHAM surgery. We found that GLX did not affect fat intake or meal patterns, but, compared with SHAM-operated controls, GLX rats had lower sugar intake, driven largely by a heightened preference for the food choice with low fat and low sugar content. These findings indicate that the GLX alone is not responsible for the elevated fat intake or meal size found in rats after 2NX, but could be solely responsible for noted reductions in sugar intake.

## 1. Introduction

Although there are multiple factors influencing food selection [1], flavor is an uncontested contributor to *what* and *how much* food is consumed [2–5]. *Flavor* describes the full oronasal sensory experience of a food and involves the integration of signals arising from the gustatory, olfactory, and trigeminal systems of which taste is a critical component [6]. Taste inputs are transmitted to the brain via branches of cranial nerves (CN) VII, IX, and X, with each nerve innervating relatively segregated fields of taste buds on the tongue, palate and laryngeal epithelium [7].

In the rat, the chorda tympani (CT) branch of CN VII innervates approximately 13% of taste buds which are found on the anterior tongue [7,8]. Another branch of CN VII, the greater superficial petrosal (GSP), innervates the approximately 16% of taste buds found on the palate [7, 8]. The glossopharyngeal (GL) nerve (CN IX) innervates the posterior tongue, which contains the largest complement of taste buds in the periphery accounting for about 56% of the total [7,8]. The remaining taste buds are found mostly in the laryngeal epithelium, innervated by the superior laryngeal branch of the vagus nerve, and are thought to be primarily involved in protection of the airways [7–9]. Decades of research suggest that the different gustatory nerves do not contribute equally to all taste functions including detection and discrimination, oromotor and somatic reflexes (called *taste reactivity*), and approach and avoidance behavior. Indeed, the differential roles of the gustatory nerves in eating-related behavior have a phylogenetic precedent as evidenced by findings of nerve-input ablation studies in fish [10–14].

Transection of gustatory nerves alone or in combination has mixed effects on taste-related behavior depending on the taste stimulus, the task, and the nerves cut [10,15–69]. Sucrose concentration-dependent a) preference in long-term two bottle tests [23,32], b) taste oromotor reflexes [32], and c) licking in a brief access test [34,51] remain unscathed by transection of the CT (CTX) alone; removal of input from another gustatory nerve (GL or GSP) is necessary to impair affective taste responsiveness to sucrose [51]. However, detection of and discrimination between salts is significantly impaired after CTX alone [16–18,20,21,28,37,46,47,49,56]. And, when the gustatory branches of CN VII (CT and GSP) are transected in combination, performance in any task requiring stimulus identification/discrimination is substantially impaired [18], at least with compounds tested so far, whereas GLX alone has little, if any, effect [19,49,50,59,60]. In contrast, GLX leads to severely attenuated elicitation of gapes and other oromotor/somatic reflexes triggered by quinine [62,67] even though GLX does not affect detection of this bitter stimulus unless combined with CTX [54,59,60]. Licking avoidance is blunted by GLX in short-term tasks in rats [40], but if the animals have had presurgical quinine exposure, then GLX must be combined with CTX to see an effect [54]. The lingual gustatory nerves are among the first to provide sensory information about food as it enters the alimentary tract. While there is extensive research investigating basic taste functions, there is surprisingly little research investigating how the sensory information provided by the gustatory nerves affects behaviors like food selection and meal patterning.

Although arguably, natural selection endowed animals with a gustatory system to make choices among complex food and fluid stimuli in the environment dependent on nutritional state, relatively little attention has been placed on the explicit effects of removal of selective peripheral taste input on eating and drinking. One study providing detailed meal pattern analysis after CTX [70] found that total intake did not change but CTX reduced the eating rate leading to increased meal duration. GLX impairs normal preference for and intake of corn oil across a range of concentrations in 24-h tests [63] but does not affect chow intake, thus leading to a change in macronutrient intake. The reduction in corn oil intake was due entirely to a decrease in corn oil meal size and not meal number. Other studies have investigated effects after transection of multiple taste nerves. A study of intake of five food items in rats after either sham surgery or CTX + GLX + transection of the pharyngeal branch of the vagus found that the neurotomized rats required more time to return to presurgical intake levels of some foods (e.g., rat chow pellets) but not others (e.g., pablum) [33]. Not surprisingly, complete transection of the gustatory branches of CNs VII and IX produces more extreme effects. After CTX + GSPX + GLX, rats decreased intake of a previously preferred sweetened milk diet. Although intake of sweetened milk increased over time postsurgically, there was a simultaneous decline in ingestion rate, suggesting the sweet milk was less palatable to the rats after surgery. Plausibly, preoperative experience may have maintained the initial postoperative sweet milk ingestion rate which declined over time as rats adapted to the denervation-induced changes in flavor perception [23]. Surprisingly, and possibly driven by similarities to the sweet milk diet, denervated rats preferred a novel sucrose solution similarly to sham-operated rats although the triple-transected rats failed to demonstrate concentration-dependent responding [23]. Importantly, these studies lacked either detailed meal patterns [33], presentation of more than two caloric choices [23, 63,70], and/or histological verification of the nerve transections [33].

We recently used our custom 5-Item Food Choice Monitor (FCM) to examine caloric intake, food choices, and meal patterns in 30 male rats after recovery from sham surgery (SHAM) or combined transection (2NX) of the GL and CT nerves, which together innervate taste buds of the tongue accounting for ~70% of the total in the periphery [71]. To our knowledge, this is the only published study conducted in a setting with constant ad libitum access to multiple foods (>2) in which detailed analyses of food choice and relative macronutrient intake were assessed on a meal-by-meal basis in animals with histologically verified gustatory nerve transections. In this study, rats had access to powdered standard rodent chow plus 4 custom rodent diets which were high (H) or low (L) in fat (F) and/or sugar (S) content but identical in protein, fiber, electrolyte, vitamin, and mineral composition. Half of each surgical group experienced the foods prior to surgery. We found effects of surgery on food choices, meal parameters, and macronutrient intake. Specifically, after histologically verified 2NX, rats consumed more of the HF choices and less of the LF choices resulting in 2NX rats having greater intake of fat and reduced intake of carbohydrates compared to SHAM rats. The 2NX rats ate at a similar rate to SHAM rats but took longer to satiate and thus consumed significantly larger meals. Since 2NX rats ate fewer meals, total daily energy intake was similar between groups. We found only minor effects of presurgical diet experience. The elevated fat intake, meal duration, and meal size suggest 2NX increased the palatability and/or reduced the satiating potency of the fatty food choices. Notably, the effects of 2NX on fat intake by rats in our choice diet model are completely opposite the result obtained in earlier work by our lab showing that GLX reduced fat intake, specifically by reducing intake of corn oil via reduced meal size [63]—suggesting that the corn oil was either *less* palatable or *more* satiating (or both). Accordingly, the primary goal of the current study was to begin quantification of changes to food choices, nutritional regulation, and specific aspects of ingestive behavior caused by selective lingual gustatory denervation, beginning with the role of the GL in food choice and meal patterns.

## 2. Methods

### 2.1. Subjects

Subjects for this experiment were 16 male Sprague-Dawley rats with an average body weight of 293.8 g one day after arrival at our facility. The rats were allowed ≥23 days to acclimate to the vivarium and handling prior to surgery or other experimental procedures and were singly housed in standard or modified (described in Section 2.3 Apparatus) standard polycarbonate tub cages with bedding in a vivarium where temperature, humidity, and the light cycle were automatically maintained. They had ad libitum access to chow (pelleted or powdered, LabDiet 5001) and deionized water and, at times indicated below, 4 additional food choices that varied in fat and sugar content (described in Section 2.4 Diets). Enrichment was provided by a Rattle A Round (Otto Environmental, Greenfield, WI), a nest, and/or multiple food choices. All procedures were approved by the Florida State University Animal Care and Use Committee.

### 2.2. Surgery

Using aseptic technique, we performed bilateral glossopharyngeal nerve transection (GLX) or sham surgery, as previously described [18, 19,23,24,39,40,49,51,54,55,59,63,72]. Briefly, the rats were anesthetized with isoflurane (≤ 5% in 1 L O_2_/min) and positioned in an earbar-less head holder. A midline incision was made to the ventral neck, and the sublingual and submaxillary salivary glands were carefully retracted on one side. The sternohyoid, omohyoid, and posterior belly of the digastric muscle were then retracted and connective tissue dissected to expose the GL, which was cut with microscissors to remove as much as possible, typically ~ 10 mm; this has been shown to discourage regeneration [72]. The procedure was repeated on the second side before the incision was closed with non-absorbable sutures. The SHAM surgery proceeded as described above except that the GL was only visualized and left undisturbed. At the time of surgery and for three days after, rats received carprofen (5 mg/kg body weight, s.c.) for control of post-surgical pain and gentamicin (8 mg/kg body weight, s.c.) to prevent infection. To encourage intake, in addition to ad libitum pelleted standard chow, the rats received a wet mash made from powdered chow and deionized water for at least three days after surgery, or until body weight returned to ≥90% pre-surgical weight. Sutures were removed 7–10 days after surgery.

### 2.3. Apparatus

No less than 10 days after surgery, rats were placed into a custom 5-Item Food Choice Monitor (FCM). The FCM is fully described elsewhere [73]. Briefly, the FCM allows continuous monitoring of intake from up to 5 food choices and licks from up to 2 fluid choices. The device consists of standard polycarbonate tub cages that have been modified to add fluid bottle supports with lick blocks that record each lick with a timestamp and a nest for the rat between them along one long side. On the opposite side is a hood with 5 separate compartments, each with a hole positioned above one of the 5 monitored food jars allowing the rat to access the foods. The load beams holding the food jars collect weight data 10 times per second and those data are assembled into 10-second bins. The positions of food jars and fluid bottles are rotated daily to prevent positional bias. Up to 8 cages are controlled by a single computer, and the data collected by the FCM are analyzed with custom software that allows the user to define the minimum meal size (1 kcal for this experiment) and the intermeal interval (900 s for this experiment).

### 2.4. Diets

The foods offered during the cafeteria diet (CAF) phase were powdered standard chow (PC) and 4 custom rodent diets (Research Diets, New Brunswick, NJ) that varied in fat and sugar content. The nutritional characteristics of all food choices are described in Table 1.

### 2.5. Histology

Seven days after the conclusion of the CAF phase of the experiment, rats were deeply anesthetized with Euthasol then transcardially perfused with 0.9% saline followed by 10% formalin. Tongues were collected and stored in formalin in the refrigerator until paraffin embedding. The posterior portion of each tongue, which contains the circumvallate papillae (**CV**), was sliced at 10 μm on a rotary microtome. Slices from the entire CV were collected and placed onto slides for staining with hematoxylin and eosin. The CVs were first qualitatively examined for tastebuds with pores and separated into those having very many tastebuds with pores (suggesting it came from an animal that had SHAM surgery) and those with few to no tastebuds with pores (suggesting it came from an animal that had GLX). Then, CVs found to have few to no taste buds with pores were re-examined and each taste bud with a pore counted. Rats with fewer than 30 taste buds, a value used previously by our laboratory [63], were considered to have successful GLX without significant regeneration (typically intact rats have >400 CV taste buds [19,55]). Tissues were coded so that the person assessing the taste buds was unaware of the group assignment. Due to the *very* apparent difference in the number of CV taste buds between SHAM and GLX rats, CVs with many tastebuds having pores were not fully counted. In this study, rats included in the GLX group had, on average, 2.71 ± 1.32 taste buds with pores in the CV. One rat that had GLX was found to have >30 taste buds and this rat was excluded from all analyses, leaving the final group sizes SHAM *n* = 8 and GLX *n* = 7.

### 2.6. Experimental timeline

**Fig. 1.**
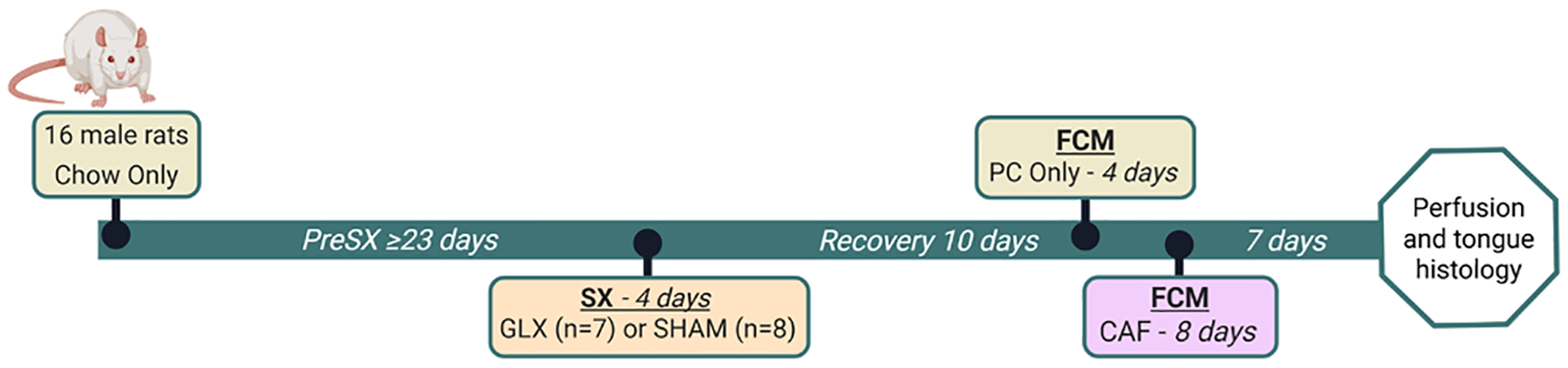
Experimental Timeline. **GLX** = glossopharyngeal nerve transection. **PreSX** = presurgery; **SX** = surgery; **FCM** = 5-Item Food Choice Monitor. **PC** = Powdered Chow. **CAF** = Cafeteria Diet. *Created in BioRender. Cawthon, C. (2025)* https://BioRender.com/7mvb35d.

Rats had at least 23 days to acclimate to our facility and handling prior to surgery. After surgery, rats were given 10 or more days to recover before additional experimental procedures began. Rats were then moved into the FCM and given only PC for 4 days followed by 8 days CAF. At the conclusion of CAF, rats were removed from the FCM and tissues were collected, as described in Section 2.5, one week later. The experimental timeline is depicted in Fig. 1.

### 2.7. Data analysis

Data from the FCM were analyzed using custom software with minimum meal size set to 1 kcal and intermeal interval defined as 900 s without intake [73]. The software determines the amount of each food consumed, meal frequency, meal size, meal duration, meal eating rate, post-meal pause, and the number of intake sources chosen during each meal (i.e., food jars and fluid bottles). Energy intake, meal pattern and food choice data were analyzed as repeated measures over the days of each phase and also by comparing the means during each phase with two-tailed, two-sample *t*-tests. To aid in the assessment of the response of rats to CAF, the average parameter value on CAF was divided by the average parameter value on PC to reveal if the difference in diets changed eating behavior (i.e., was CAF/PC different from 1?) and if the direction and magnitude of change was similar between the groups. Within groups, the CAF/PC ratios were tested for difference from 1.0 with one-sample *t*-tests, and two-sample *t*-tests were used to compare CAF/PC ratios between groups. Data are presented as mean ± SE and in all analyses, there are 8 SHAM and 7 GLX rats. All data were analyzed with SYSTAT 13 (Grafiti, Palo Alto, CA) and, in the case of mixed multifactor ANOVAs (Treatment X Day), Bonferroni-corrected pairwise comparisons were conducted when indicated (α=0.05).

## 3. Results

**Fig. 2.**
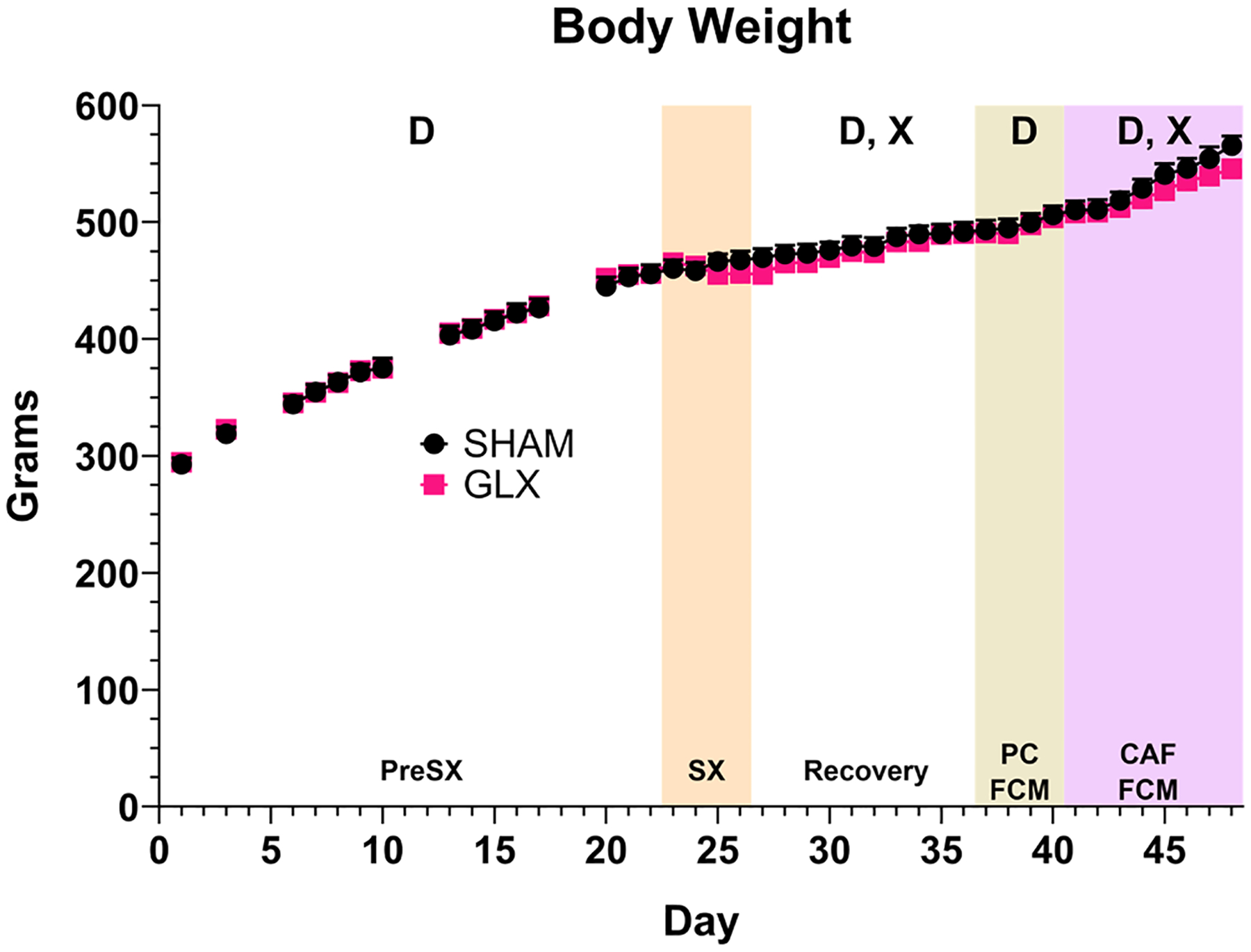
Body Weight. Body weight of rats that had SHAM surgery (black circles, *n* = 8) or GLX (pink squares, *n* = 7) shown as mean ± SE. ANOVAs were run for each phase (pre-surgery, **PreSX**, Days 1–22; surgery (**SX**, orange shading), Days 23–26; Recovery, Days 27–36; PC in the FCM (tan shading), Days 37–40; and CAF in the FCM (purple shading), Days 41–48) and significant effects of Surgery (S), Day (D), and Surgery x Day interaction (X) are indicated in each phase. Details of all statistical outcomes may be found in Table 2.

The body weights of rats that had GLX did not differ from SHAM rats before or during the surgical period (Fig. 2, Table 2). A significant interaction between surgery and day during the Recovery period resulted from GLX rats losing more weight postoperatively compared with SHAM controls. During CAF, a surgery x day interaction reflected a different rate of body weight gain between groups. These interactions prompted pairwise comparisons of each of the 10 Recovery and 8 CAF days, but the groups did not differ in body weight on any day. Still, the difference in body weight trajectory during CAF suggests a behavioral or metabolic difference between the groups during that phase, albeit slight.

**Fig. 3.**
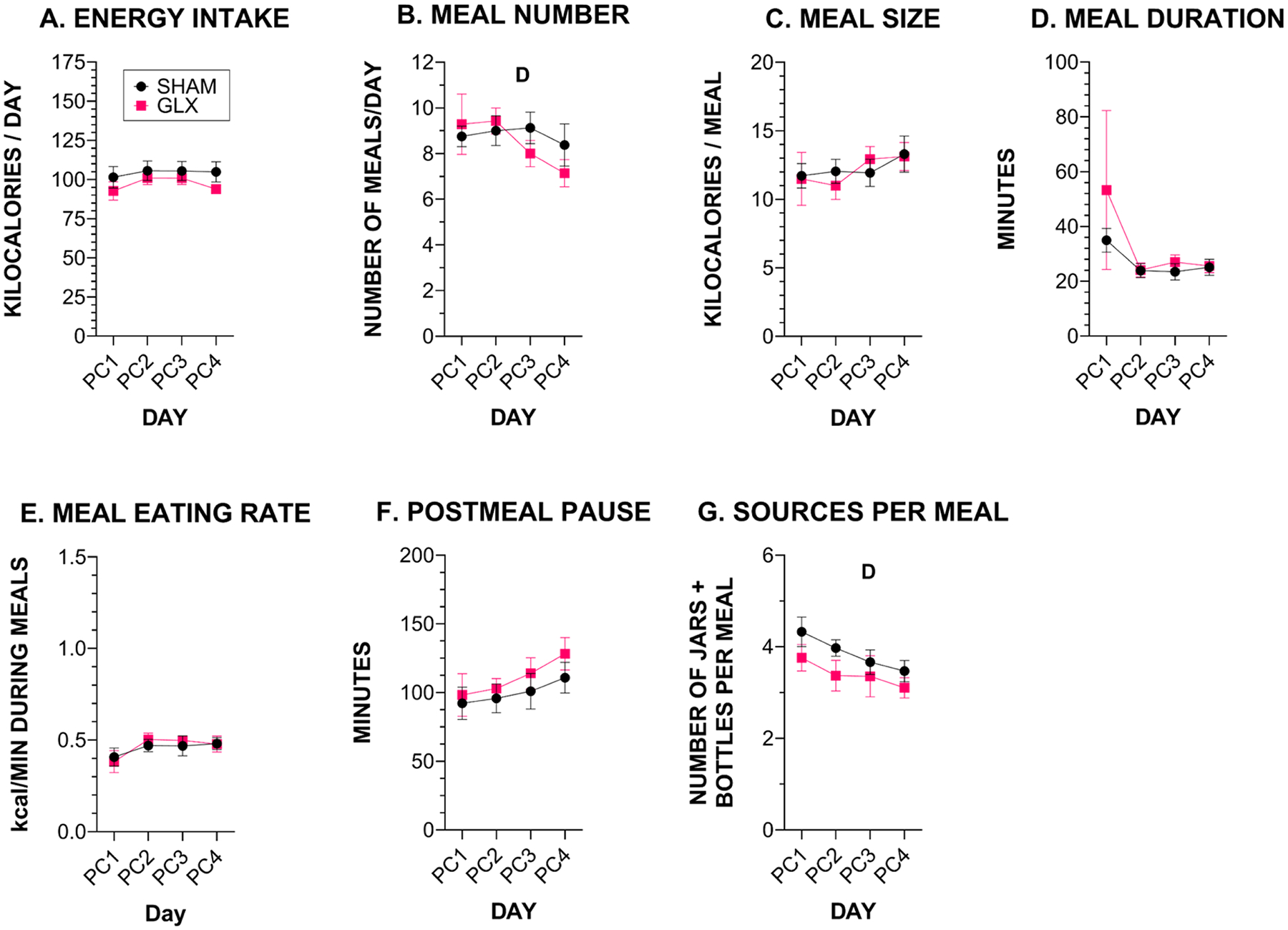
Energy intake and meal characteristics for each day that rats (SHAM *n* = 8, GLX *n* = 7) were offered only PC, shown as mean ± SE. **A**. Daily energy intake. **B**. Number of meals/day. **C**. Average meal size in kcal **D**. Meal duration. Eating rate during meals as **E**. kcal/minute. **F**. Duration of pause between meals. **G**. Number of intake sources (food jars + fluid bottles) during meals. *D* = significant effect of Day. Details of all statistical outcomes may be found in Table 3.

Looking at the 4-d PC phase, total energy intake did not differ between the groups, and rats in both groups achieved their intake with a similar number of meals of similar size (Fig. 3, Figure S4, Table 3, Table S4). Rats in both groups spent a similar amount of time consuming each meal and, therefore, eating rate did not differ. The correspondence in eating rate suggests rats in both groups were similarly motivated to consume PC. It appears that rats learned during the PC phase that all jars contained the same food, as the number of intake sources per meal decreased in both groups over the 4 days of the PC phase (4.3 ± 0.3 to 3.5 ± 0.2 for SHAM and 3.7 ± 0.3 to 3.2 ± 0.2 for GLX). It is possible that the interruption to their behavior from 2 h without food access could have driven consumption of a first meal that differed from the whole-day averages. Therefore, in addition to looking at meal pattern measures as the average for each 22-h recording period, we also assessed the characteristics of the first meal in each recording period (Figure S1, Table S1). We did not find significant differences between the groups on first meal characteristics including latency to begin first meal, meal size, meal duration, meal eating rate, number of jars eaten from or period of no intake after the first meal during the PC phase.

**Fig. 4.**
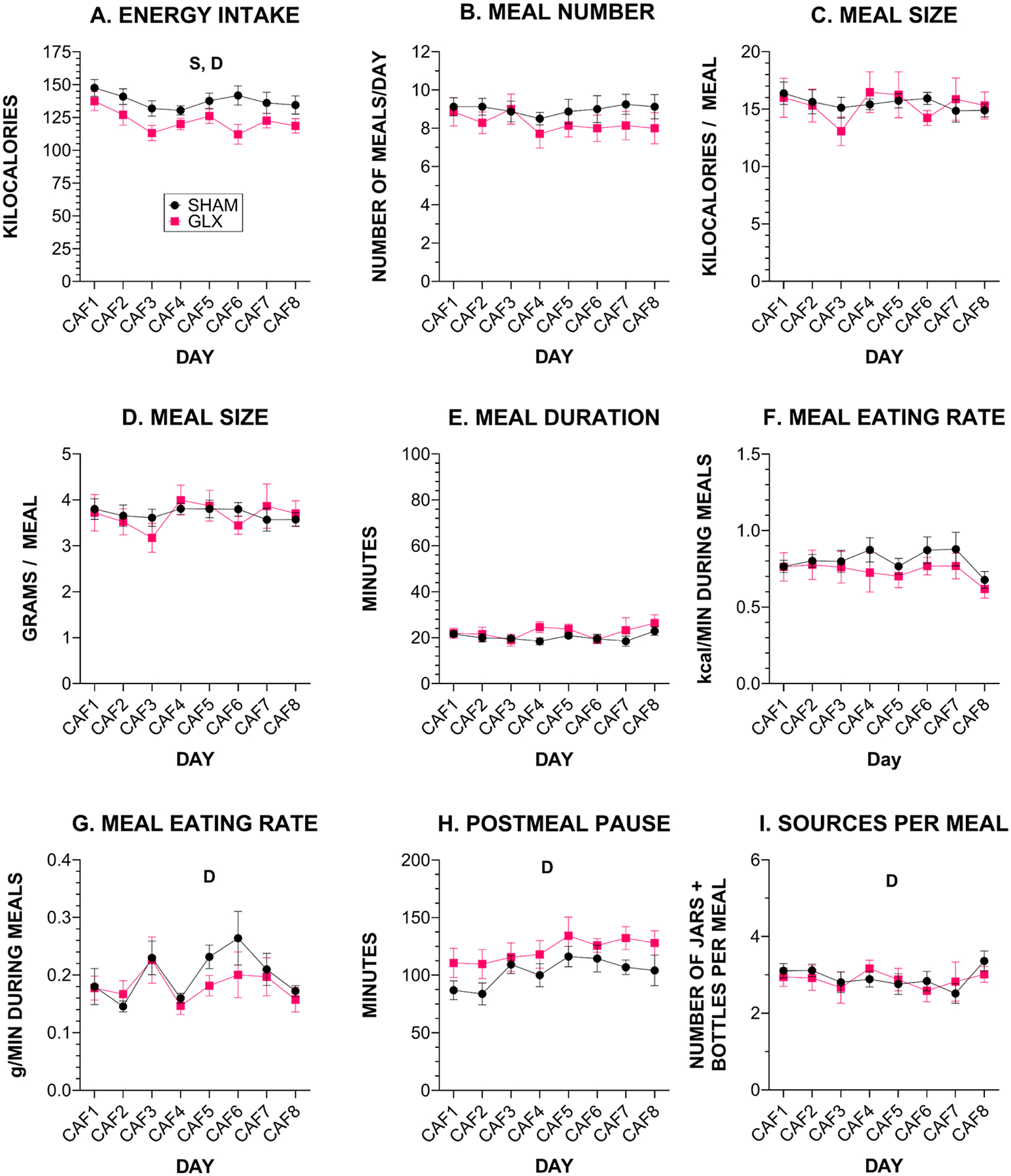
Energy intake and meal characteristics for each day that rats were offered CAF shown as mean ± SE (SHAM *n* = 8, GLX *n* = 7. **A**. Daily energy intake. **B**. Number of meals/day. **C**. Meal size in kcal **D**. Meal size in grams **E**. Meal duration. Eating rate during meals as **F**. kcal/minute and **G**. grams/minute. **H**. Duration of pause between meals. **I**. Number of intake sources (food jars + fluid bottles) during meals. *S* = significant effect of surgery; *D* = significant effect of Day. Details of all statistical outcomes may be found in Table 4.

During the 8-day CAF phase, GLX rats ate fewer kcal than rats that had SHAM surgery (average intake 137.6 ± 2.2 kcal vs. 122.1 ± 3.6 kcal, Fig. 4, Figure S4, Table 4, Table S4) and this corresponds with the slower weight gain trajectory during the CAF phase in rats that had GLX (Fig. 2, Table 2). There was no significant effect on the number or size of meals during the CAF phase, suggesting there may have been small and idiosyncratic changes across multiple meal parameters driving the reduced energy intake by the GLX rats (Fig. 4, Figure S4, Table 4, Table S4). As with PC, we did not find significant effects of surgery on average meal parameters or for the first meals (Figure S2, Table S2) of each 22-h recording session during CAF.

**Fig. 5.**
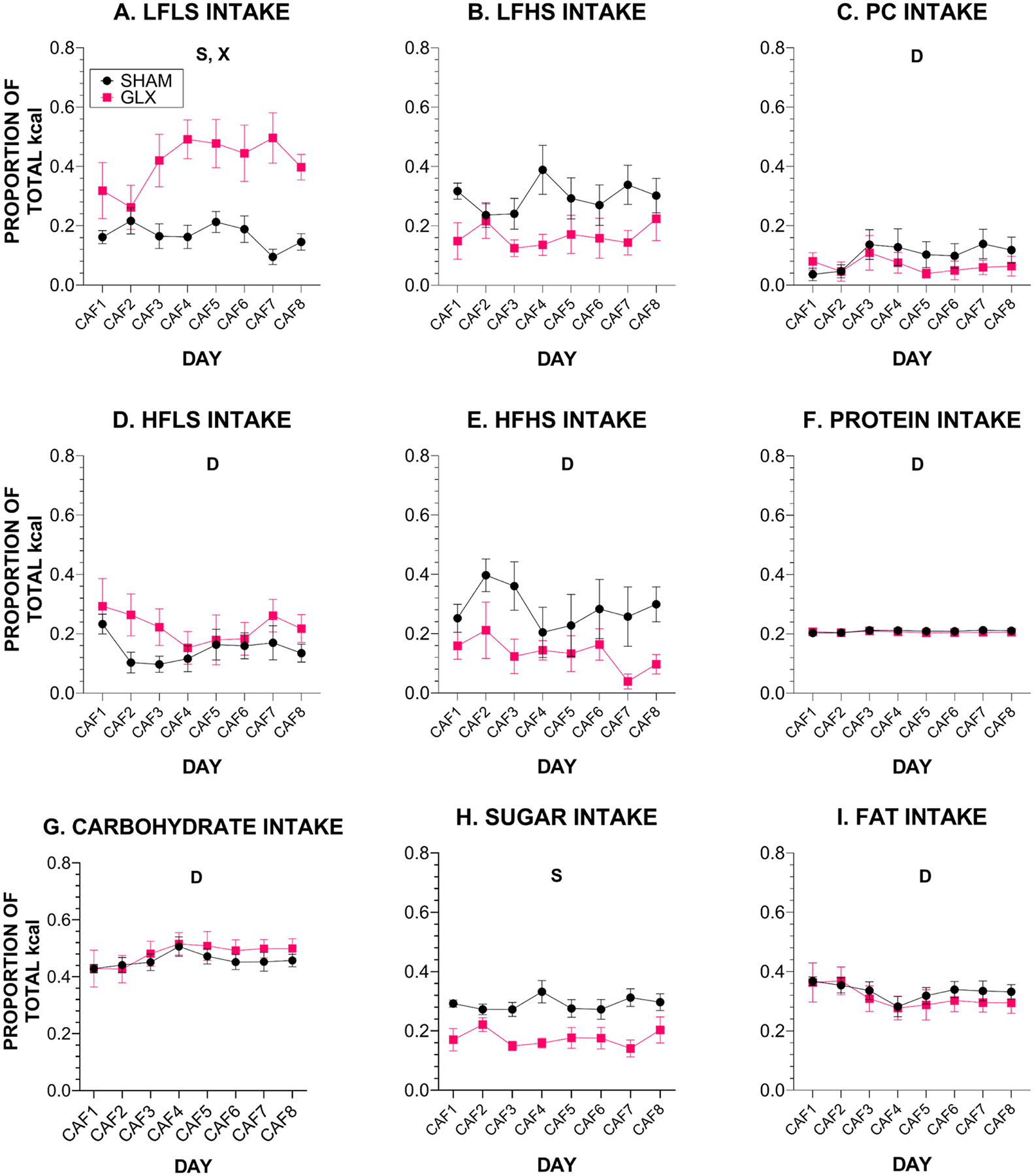
Proportion of kcal intake from individual foods, macronutrients, and sugar during CAF shown as mean ± SE proportion of total energy intake (SHAM *n* = 8, GLX *n* = 7). Proportion of total energy intake from **A**. LFLS; **B**. LFHS; **C**. PC; **D**. HFLS; **E**. HFHS; **F**. Protein; **G**. all Carbohydrate; **H**. Sugar; and **I**. Fat. *S* = significant effect of Surgery; *D* = significant effect of Day; *X* = significant surgery x day interaction. Details of all statistical outcomes may be found in Table 5.

While there were minimal differences between surgical groups for meals as a whole, distinctions emerged in the analysis of the food choices that comprised each meal. When we looked at intake of the individual food choices during CAF, we found that, compared with rats that had SHAM surgery, GLX rats consistently consumed more of the LFLS choice (average 41.3 ± 6.3% vs. 16.8 ± 2.6%) with trends for choosing less of the LFHS (average 16.6 ± 3.8% vs. 29.8 ± 5.1%, *p* = 0.061) and HFHS (average 13.4 ± 3.1% vs. 28.5 ± 7.6%, *p* = 0.093) (Fig. 5, Figure S5, Table 5, Table S4). Overall, this resulted in GLX rats consuming less sugar than SHAM controls (average 17.5 ± 2.0% vs. 29.1 ± 2.2%). Fat, total carbohydrate, and protein intake did not differ between the groups (Fig. 5, Figure S5, Table 5, Table S4). Food and macronutrient intake during the first meal of each 22-h recording period (Figure S3, Table S3) generally aligned with intake for the entire day (Fig. 5, Table 5).

To quantify differences in the responses to PC and CAF and determine if diet-induced behavioral differences were similar between GLX and SHAM rats, we assessed differences in average energy intake and meal parameters over all days of each diet using the ratio CAF/PC. It is important to clarify that the CAF/PC ratio is designed to assess behavioral adaptation; it is not intended to replace the analyses of absolute group differences in intake. Rather, it is intended to interpretively supplement them, by controlling for differences in total caloric intake. We did not find a significant difference between groups in the CAF/PC ratio for any parameter and further found that rats in both groups changed their behavior similarly on CAF as compared to PC with only two exceptions (Figure S5, Figure S6, Table S4, Table S5). Rats that had GLX increased their postmeal pause on CAF vs. PC and did not significantly decrease their meal duration whereas rats that had SHAM did not have a change in postmeal pause but reduced their meal duration; these changes were minor and did not result in any difference in CAF/PC ratios between groups (Figure S6, Table S4, Table S5).

## 4. Discussion

The primary goal of the study presented here was to quantify changes to food choices, nutritional regulation, and specific meal-related aspects of ingestive behavior caused by GLX. We chose to explore the role of the GLX since our earlier findings in rats that had combined transection of both lingual taste nerves (2NX) [71] identified a different response to fat compared to historical results of GLX alone [63]. Here, we found that GLX did not produce differences in meal-taking behaviors vs SHAM rats when PC was the only food choice. When offered choices varying in fat and sugar content, GLX led to the ingestion of less sugar, a reduction in total energy intake, and a slower weight gain. Previously we found that 2NX profoundly elevated fat intake and, to a lesser degree, reduced sugar intake, with the difference in sugar intake being somewhat greater in animals that did not experience the CAF choices prior to surgery [71]. The results presented here suggest that the GL is responsible for the effects we found on sugar intake in our prior work, but the GL does not play a singular role in the changes to fat intake resulting from 2NX. It is possible that the previous effect on fat intake was driven by transection of the chorda tympani nerve or that both nerves must be cut to produce the effect. Future experiments could investigate how chorda tympani transection alone affects food intake and meal patterns in rats offered these food choices.

Earlier work by our lab found that intake of a corn oil emulsion was reduced in rats that had GLX [63], suggesting GLX reduced preference for fat. Here, using a 5-choice cafeteria diet with solid foods varying in fat and sugar content, we found that GLX did not affect fat intake. When our prior work revealed that 2NX increased fat intake on this same 5-choice diet, we posited that our findings could be driven by alterations in the response to the form or energy density of the food choices or that a modulatory effect of the CT led to the apparently opposite findings between the corn oil study [63] and our 2NX results [71]. Now, with GLX alone (i.e., when these rats were missing the same sensory information as the rats in the corn oil study [63]), we find no effect of GLX on fat intake, once again differing from the original work using corn oil emulsions. This finding suggests that a characteristic of the food choices (e.g., liquid vs. solid, lower energy density vs. higher energy density, simple vs. complex) or food environment (2 choices vs 5 choices) influences how GLX affects fat intake. We would note, however, that we have not eliminated potential modulation by the CT as a key component of our earlier finding that 2NX increases fat intake and meal size. Further testing with food choices that vary in form and energy density could be conducted to better determine what role food characteristics may play in post-GLX food choice.

Here, we found that GLX produced changes in food choice resulting in reduced sugar intake, which we also found when GLX was combined with CTX [71] and in agreement with earlier findings that 2NX reduced sucrose preference in 48-h 2-bottle preference tests [74]. Historical studies of the effects of GLX on basic responses to sucrose find that GLX alone does not alter ingestive or aversive taste reactivity to sucrose [32, 62], has little effect on concentration-dependent [51] and non-concentration dependent [75] brief access licking, does not prevent the conditioning of a taste aversion to sucrose [24], and, although GLX was unverified, does not reduce intake of low sucrose concentrations in mice [61] or rats [76], suggesting suprathreshold responsiveness to sucrose is at least not significantly impaired by GLX. That said, the effects of GLX on detection of sugars and starches should be specifically tested using rigorous psychophysical methods. Nonetheless, it seems unlikely that our GLX rats reduced their sugar intake due to a complete inability to detect sucrose or because they found sucrose less palatable, but it is possible that even minor deficits in sucrose intensity induced by GLX could affect food choices on CAF. Further support for a lack of a GLX effect on palatability comes from the absence of a difference in meal eating rates, with the caveat that interpretation of the mixed-composition meal rate is complex. Still, consistent with the results of this study, earlier research from our lab found that GLX reduced intake of glucose, leading to lower energy intake without clearly identifying a single meal parameter driving the effect [63]. Importantly, the conclusion from studies using solutions of pure compounds may not be directly transferrable to our CAF with nutritionally complex options.

The LFLS and LFHS food choices have identical energy densities (3.79 kcal/g) and are both dry powders, but the texture of these foods differ in that they resemble their most abundant ingredients (63% cornstarch or 66.5% sucrose). However, Sclafani et al. [77], found powdered sucrose was preferred to powdered corn starch and this should correspond well to the textures of the food choices presented here. Indeed, as the results of Sclafani et al., would predict, our SHAM operated rats averaged 16.8 ± 1.3% of total kcal from the LFLS choice and 29.8 ± 2.1% from the LFHS option while rats that had GLX consumed, on average, 37.6 ± 2.9% LFLS and 16.0 ± 1.8% LFHS. Other research shows that rats can detect corn starch [78], prefer it to cellulose, [77] and in short-term tests, prefer it to polycose [77]. Furthermore, there is evidence that intact rats can discriminate corn starch from sucrose, at least in liquid form [78]. Since GLX does not appear to alter the behavioral responses to sucrose [24,32,61,76] and sucrose is preferred to cornstarch [77], one possibility is that GLX alters the perception of cornstarch in a way that increases its palatability compared to sucrose. Despite our SHAM rats behaving as predicted, it is important to consider that a key difference between the study by Sclafani et al., and this study is that our rats had more than two choices and we and others have previously found that the available choices influence intake-based preference [79,80]. It is notable that, although not quite significant, there was a trend for GLX rats to consume less of both HS options so it is possible that in the context of the available choices, the high sugar content and grainier texture of these foods was less palatable or the smoother textures of the LS choices was more palatable. Our approach does not allow us to determine whether we have in fact found an effect of GLX on corn starch intake/preference or an instance where food choices are influenced by the entire food environment. Nor are we able to determine if downstream changes (i.e., nucleus of the solitary tract, parabrachial nucleus) occurred or how they could have influenced changes in behavior, but evidence suggests that damage to gustatory nerves alters the anatomy of central structures [e. g., 81,82]. Finally, it is possible that additional subjects would have allowed for better ability to detect the modest effects we found on the intake of the individual HS options. However, we have observed idiosyncratic behavior with CAF choices in the past ([e.g., 79] and suggest the more robust effects at the macronutrient level are the key story. The effects of GLX on preference for and detection of cornstarch could be addressed in future research projects.

Although we have considered possibilities in the preceding paragraph, the question of exactly how GLX may have altered carbohydrate choice remains unanswered; the choice of cornstarch over sucrose as seen here would not be predicted from the literature. It is tempting to speculate about the mechanism. Perhaps some aversive property (e.g., textural, chemical impurity) of the cornstarch becomes less detectable after GLX. Or perhaps the behavior was driven by enhanced CT function after GLX, as has been suggested [e. g., 83,84]. Further speculation might consider a potential role of the gut or oral microbiota. To our knowledge, the possibility that GLX alone would change the microbiome composition has not been investigated. If GLX influenced microbiota composition either directly or indirectly as a function of the foods ingested [e. g., 85,86], this could, in turn, potentially contribute to nutrient selection, given there is evidence the microbiome can influence behavior [reviewed in 87]. All of these speculations are worthy of further scrutiny in future experiments.

## 5. Conclusion

Here we aimed to determine the role of the GL in the striking changes to food choice, meal patterns, and macronutrient intake that we found after 2NX (combined transection of the GL and CT). The results presented here indicate that GLX may account for the reduction in sugar intake we found after 2NX but no indication of an effect on fat intake or meal patterns was found in the present study. This suggests that our findings from 2NX showing changes in fat intake and meal patterns result from CTX or that 2NX is required to produce those effects. Future studies should address the effects of CTX alone on choices and meal patterns with this CAF. Additionally, a rigorous investigation of the effects of GLX on detection and selection of carbohydrates is warranted based on our findings here.

## Supplementary Material

1

Supplementary material associated with this article can be found, in the online version, at doi:10.1016/j.physbeh.2026.115268.

## Figures and Tables

**Table 1:** Diet Characteristics.

Choice	kcal/g	Carbohydrate		FAT	PRO
		SUG	NON		
Powdered Chow (**PC**)	3.35	9.2	48.3	13.6	28.9
Low-fat Low-sugar (**LFLS**)	3.79	4.0	63.0	13.0	20.0
Low-fat High-sugar (**LFHS**)	3.79	66.5	0.5	13.0	20.0
High-fat Low-sugar (**HFLS**)	5.41	4.0	6.0	70.0	20.0
High-fat High-sugar (**HFHS**)	4.85	24.8	0.6	54.6	20.0

Macronutrients in cafeteria diet (**CAF**) choices shown as% kcal. **SUG** = Sugar. **NON** = Non-sugar carbohydrate. **PRO** = Protein.

**Table 2:** Body Weight Statistics by Phase.

MEASURE	PHASE	EFFECT OF SURGERY	EFFECT OF DAY	SURGERY × DAY INTERACTION
**Body Weight**	**PRESX**	F (1, 13) = 0.010, *p* = 0.920	**F (14, 182) = 1250.30, *p* < 0.001**	F (14, 182) = 0.497, *p* = 0.933
	**SX**	F (1, 13) = 0.005, *p* = 0.947	F (3, 39) = 0.753, *p* = 0.527	F (3, 39) = 0.568, *p* = 0.453
	**RECOVERY**	F (1, 13) = 1.015, *p* = 0.332	**F (9, 117) = 50.402, *p* < 0.001**	**F (9, 117) = 2.299, *p* = 0.020**
	**PC**	F (1, 13) = 0.248, *p* = 0.627	**F (3, 39) = 67.080, *p* < 0.001**	F (3, 39) = 1.603, *p* = 0.204
	**CAF**	F (1, 13) = 0.841, *p* = 0.376	**F (7, 91) = 193.416, *p* < 0.001**	**F (7, 91) = 6.829, *p* = 0.004**

Bolded text = *p* < 0.05. PRESX = Presurgery; SX = Surgery.

**Table 3:** Energy Intake and Meal Characteristics (PC Days).

MEASURE	EFFECT OF SURGERY	EFFECT OF DAY	SURGERY x DAY INTERACTION
**Energy Intake**	F (1, 13) = 1.157, *p* = 0.302	F (3, 39) = 1.514, *p* = 0.226	F (3, 39) = 0.415, *p* = 0.743
**Meal Number**	F (1, 13) = 0.157, *p* = 0.699	**F (3, 39) = 3.199, *p* = 0.034**	F (3, 39) = 1.765, *p* = 0.170
**Meal Size (kcal)**	F (1, 13) = 0.007, *p* = 0.932	F (3, 39) = 1.778, *p* = 0.167	F (3, 39) = 0.498, *p* = 0.686
**Meal Duration**	F (1, 13) = 0.619, *p* = 0.446	F (3, 39) = 1.894, *p* = 0.147	F (3, 39) = 03,376, *p* = 0.771
**Eating Rate (kcal/min)**	F (1, 13) = 0.045, *p* = 0.835	F (3, 39) = 2.823, *p* = 0.051	F (3, 39) = 0.279, *p* = 0.840
**Postmeal Pause**	F (1, 13) = 0.909, *p* = 0.358	F (3, 39) = 2.334, *p* = 0.089	F (3, 39) = 0.143, *p* = 0.933
**Number Intake Sources /Meal**	F (1, 13) = 1.837, *p* = 0.198	F (3, 39) = 5.360, *p* = 0.003	F (3, 39) = 0.275, *p* = 0.843

**Bold** = *p* < 0.05.

**Table 4:** Energy Intake and Meal Characteristics CAF.

MEASURE	EFFECT OF SURGERY	EFFECT OF DAY	SURGERY x DAY INTERACTION
**Energy Intake**	**F (1, 13) = 6.246, *p* = 0.027**	**F (7, 91) = 3.219, *p* = 0.004**	F (7, 91) = 0.839, *p* = 0.558
**Meal Number**	F (1, 13) = 1.293, *p* = 0.276	F (7, 91) = 0.690, *p* = 0.680	F (7, 91) = 0.421, *p* = 0.887
**Meal Size (kcal)**	F (1, 13) = 0.030, *p* = 0.866	F (7, 91) = 0.929, *p* = 0.488	F (7, 91) = 0.713, *p* = 0.661
**Meal Size (g)**	F (1, 13) = 0.027, *p* = 0.871	F (7, 91) = 1.056, *p* = 0.399	F (7, 91) = 0.701, *p* = 0.671
**Meal Duration**	F (1, 13) = 0.875, *p* = 0.367	F (7, 91) = 1.778, *p* = 0.101	F (7, 91) = 0.922, *p* = 0.494
**Eating Rate (kcal/min)**	F (1, 13) = 0.805, *p* = 0.386	F (7, 91) = 1.676, *p* = 0.125	F (7, 91) = 0.305, *p* = 0.950
**Eating Rate (g/min)**	F (1, 13) = 0.814, *p* = 0.383	F (7, 91) = 3.177, *p* = 0.005	F (7, 91) = 0.589, *p* = 0.763
**Postmeal Pause**	F (1, 13) = 2.879, *p* = 0.114	F (7, 91) = 3.507, *p* = 0.002	F (7, 91) = 0.427, *p* = 0.883
**Number of Intake Sources /Meal**	F (1, 13) = 0.025, *p* = 0.877	F (7, 91) = 2.131, *p* = 0.048	F (7, 91) = 0.943, *p* = 0.478

**Bold** = *p* < 0.05.

**Table 5:** Foods and Macronutrients: Proportion of Total Energy Intake.

MEASURE		EFFECT OF SURGERY	EFFECT OF DAY	SURGERY x DAY INTERACTION
	**LFLS**	**F (1, 13) = 13.939, *p* = 0.003**	F (7, 91) = 1.925, *p* = 0.074	**F (7, 91) = 3.643, *p* = 0.002**
	**LFHS**	F (1, 13) = 4.188, *p* = 0.061	F (7, 91) = 0.887, *p* = 0.520	F (7, 91) = 1.689, *p* = 0.121
	**PC**	F (1, 13) = 0.521, *p* = 0.483	**F (7, 91) = 2.688, *p* = 0.014**	F (7, 91) = 1.674, *p* = 0.125
	**HFLS**	F (1, 13) = 1.641, *p* = 0.223	**F (7, 91) = 2.133, *p* = 0.048**	F (7, 91) = 0.905, *p* = 0.506
**% of kcal Intake from**	**HFHS**	F (1, 13) = 3.294, *p* = 0.093	**F (7, 91) = 2.446, *p* = 0.024**	F (7, 91) = 1.182, *p* = 0.321
	**Protein**	F (1, 13) = 0.521, *p* = 0.483	**F (7, 91) = 2.688, *p* = 0.014**	F (7, 91) = 1.674, *p* = 0.125
	**Carb**	F (1, 13) = 0.305, *p* = 0.590	**F (7, 91) = 3.485, *p* = 0.002**	F (7, 91) = 0.599, *p* = 0.755
	**Sugar**	**F (1, 13) = 15.027, *p* = 0.002**	F (7, 91) = 0.791, *p* = 0.597	F (7, 91) = 1.789, *p* = 0.099
	**Fat**	F (1, 13) = 0.227, *p* = 0.642	**F (7, 91) = 3.751, *p* = 0.001**	F (7, 91) = 0.457, *p* = 0.863

**Bold** = *p* < 0.05.

## Data Availability

Source data for this study are openly available at: https://doi.org/10.5061/dryad.dncjsxmdf.
