# Supplementary material for: Transection of the glossopharyngeal nerve reduces energy and sugar intake but does not affect fat intake or meal patterns in rats offered a palatable cafeteria diet
Source: Physiol Behav. Author manuscript; Available in PMC 2026 May 27. (PMC13214985; doi:10.1016/j.physbeh.2026.115268)
Supplement: 1 [file NIHMS2178245-supplement-1.pdf]

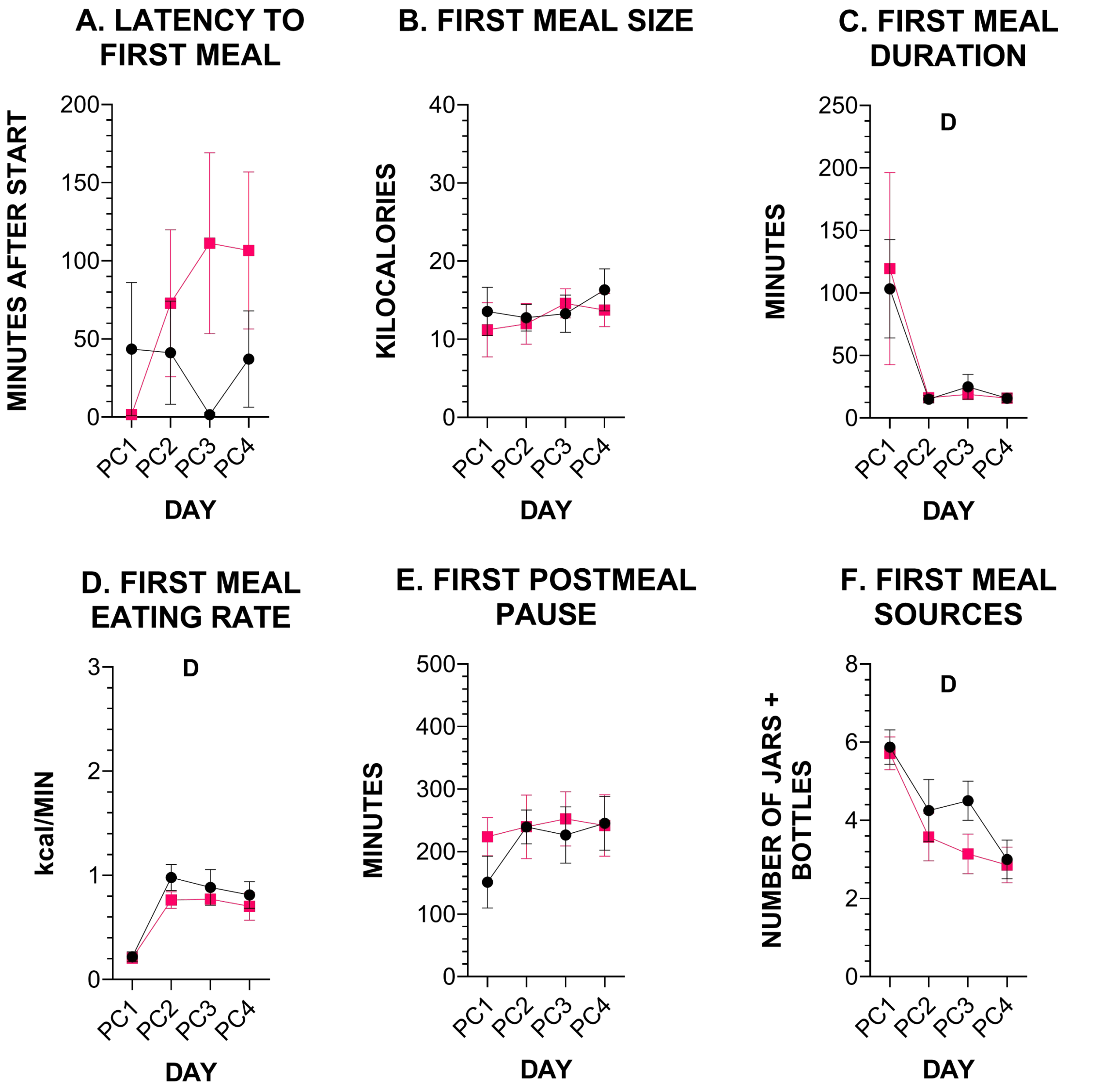

Figure S1. First meal characteristics during PC shown as mean±SE (SHAM n=8, GLX n=7). **A**. Latency to first meal initiation as seconds from session start. **B**. First meal size in kcal **C**. First meal duration; First meal eating rate as **D**. kcal/min; **E**. First postmeal pause; **F.** Number of intake sources (food jars + fluid bottles) during first meal. D = significant effect of Day. Details of all statistical outcomes may be found in **Table S1**.

**Table S1.** First Meal Characteristics PC			
**Latency to First Meal**	F (1, 13) = 1.631, p = 0.224	F (3, 39) = 0.736, p = 0.537	F (3, 39) = 1.763, p = 0.170
**First Meal Size (kcal)**	F (1, 13) = 0.290, p = 0.599	F (3, 39) = 0.158, p = 0.924	F (3, 39) = 0.299, p = 0.876
**First Meal Duration**	F (1, 13) = 0.021, p = 0.888	**F (3, 39) = 4.830, p = 0.006**	F (3, 39) = 0.049, p = 0.986
**First Meal Eating Rate (kcal/min)**	F (1, 13) = 1.437, p = 0.252	**F (3, 39) = 17.881, p < 0.001**	F (3, 39) = 0.330, p = 0.804
**First Postmeal Pause**	F (1, 13) = 0.498, p = 0.493	F (3, 39) = 0.898, p = 0.451	F (3, 39) = 0.389, p = 0.762
**First Meal Intake Sources**	F (1, 13) = 0.951, p = 0.347	**F (3, 39) = 17.841, p < 0.001**	F (3, 39) = 1.004, p = 0.401
**Bold** = p < 0.05.			

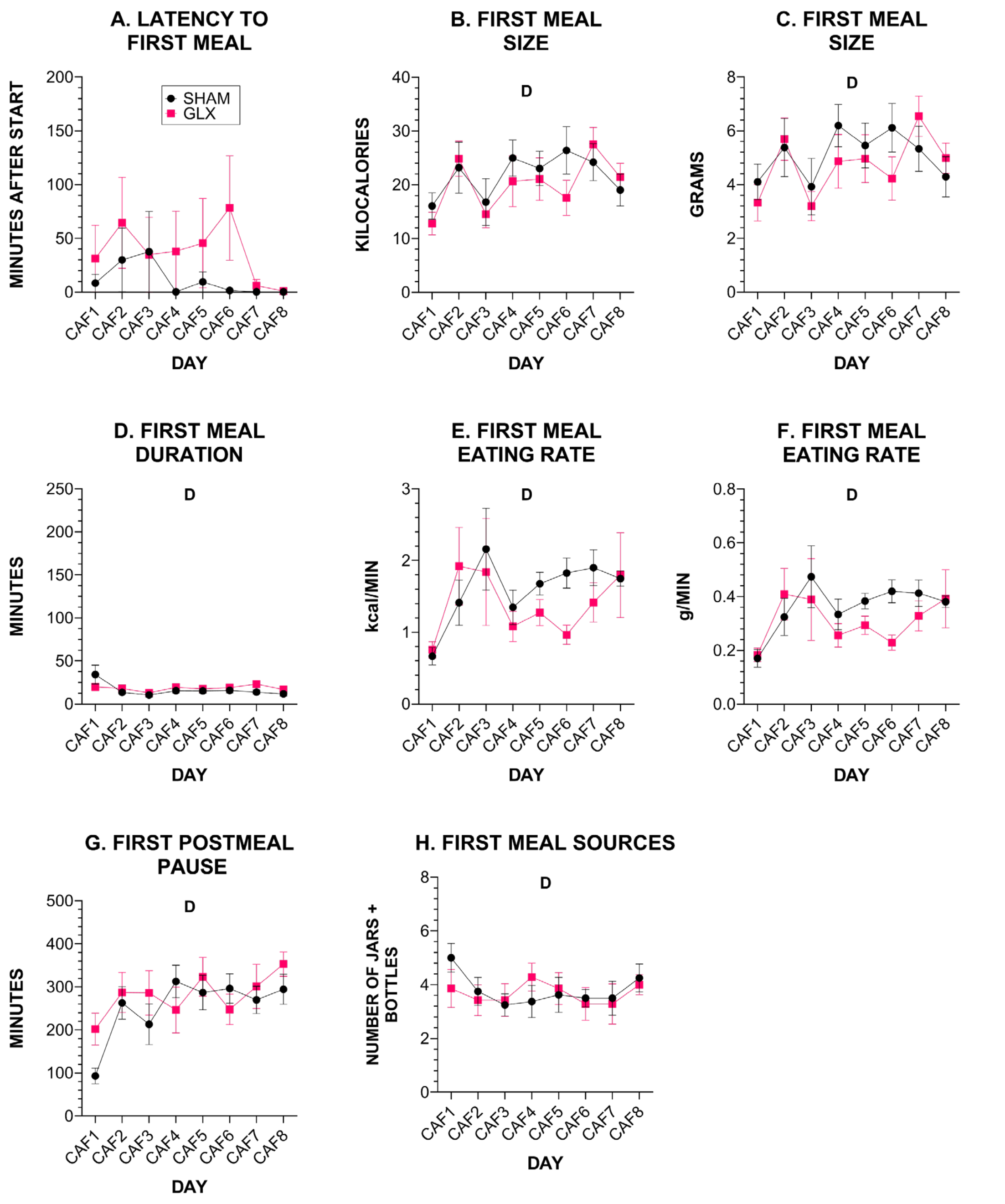

Figure S2. First meal characteristics during CAF shown as mean±SE (SHAM n=8, GLX n=7). **A**. Latency to first meal initiation as seconds from session start. **B**. First meal size in kcal and **C**. grams; **D**. First meal duration; First meal eating rate as **E**. kcal/min and **F**. grams/min; **G**. First postmeal pause; **H.** Number of intake sources (food jars + fluid bottles) during first meal. D = significant effect of Day. Details of all statistical outcomes may be found in **Table S2**.

**Table S2.** First Meal Characteristics CAF			
**MEASURE**	**EFFECT OF SURGERY**	**EFFECT OF DAY**	**SURGERY x DAY INTERACTION**
**Latency to First Meal**	F (1, 13) = 1.251, p = 0.284	F (7, 91) = 1.214, p = 0.303	F (7, 91) = 0.733, p = 0.644
**First Meal Size (kcal)**	F (1, 13) = 0.078, p = 0.784	**F (7, 91) = 3.088, p = 0.006**	F (7, 91) = 1.016, p = 0.425
**First Meal Size (g)**	F (1, 13) = 0.063, p = 0.806	**F (7, 91) = 2.599, p = 0.017**	F (7, 91) = 1.106, p = 0.366
**First Meal Duration**	F (1, 13) = 0.663, p = 0.430	**F (7, 91) = 2.435, p = 0.025**	F (7, 91) = 1.520, p = 0.170
**First Meal Eating Rate (kcal/min)**	F (1, 13) = 0.690, p = 0.421	**F (7, 91) = 3.076, p = 0.006**	F (7, 91) = 0.861, p = 0.540
**First Meal Eating Rate (g/min)**	F (1, 13) = 1.156, p = 0.302	**F (7, 91) = 2.824, p = -0.010**	F (7, 91) = 0.873, p = 0.531
**First Postmeal Pause**	F (1, 13) = 0.577, p = 0.461	**F (7, 91) = 5.114, p < 0.001**	F (7, 91) = 1.570, p = 0.154
**First Meal Intake Sources**	F (1, 13) = 2.270, p = 0.156	**F (7, 91) = 2.373, p = 0.028**	F (7, 91) = 1.250, p = 0.284
**Bold =** p < 0.05			

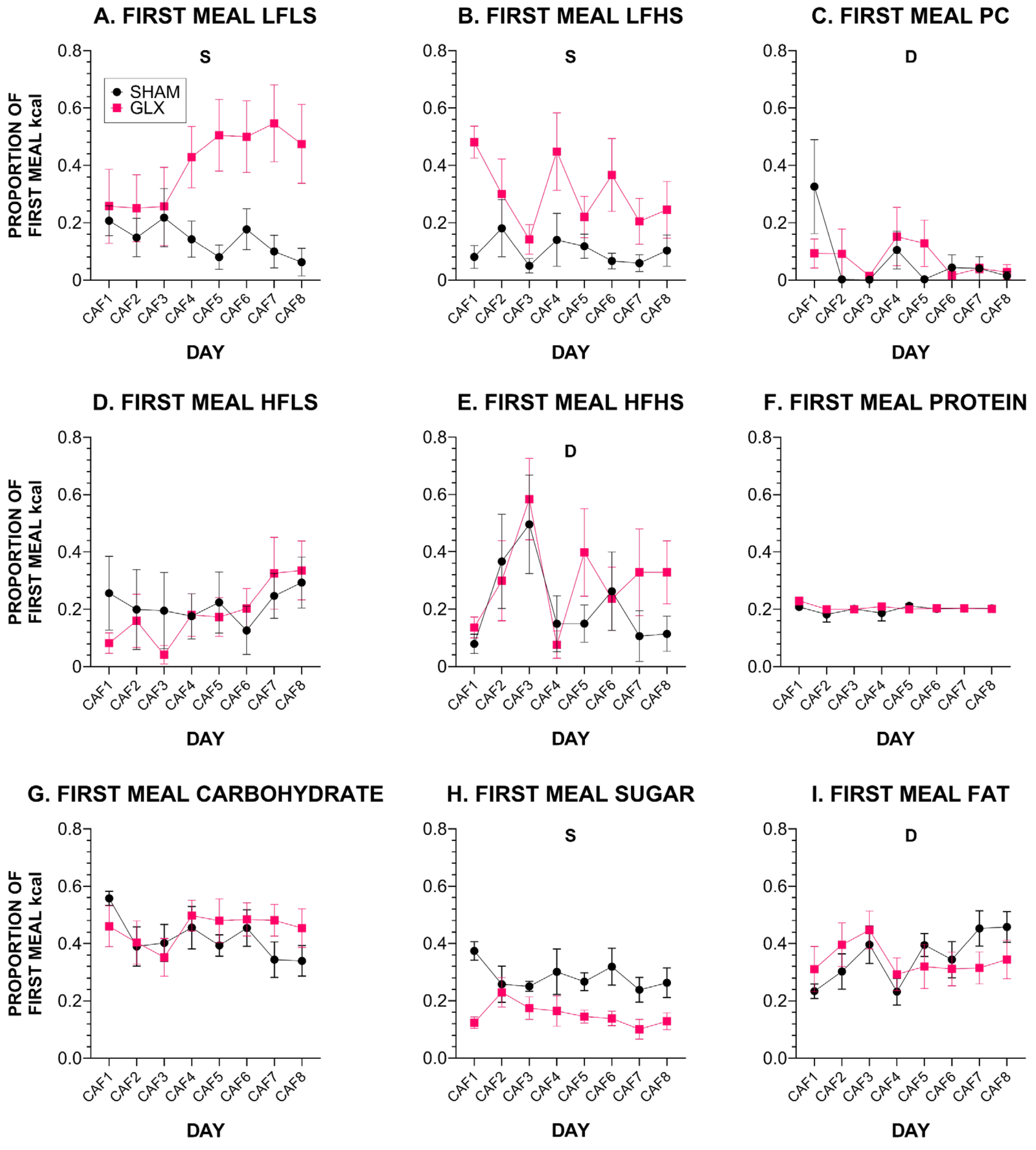

Figure S3. First meal intake of individual foods, macronutrients, and sugar during CAF shown as mean±SE proportion of total energy intake (SHAM n=8, GLX n=7). Proportion of total first meal energy intake from **A.** LFLS; **B.** LFHS; **C.** PC; **D.** HFLS; **E.** HFHS; **F.** Protein; **G.** all Carbohydrate; **H.** Sugar; and **I.** Fat. S = significant effect of Surgery; D = significant effect of Day; X = significant surgery x day interaction. Details of all statistical outcomes may be found in **Table S3**.

**Table S3. First Meal** Foods and Macronutrients: Proportion of Total Energy Intake				
**MEASURE**		**EFFECT OF SURGERY**	**EFFECT OF DAY**	**SURGERY x DAY INTERACTION**
**% of kcal Intake During First Meal from**	**LFLS**	**F (1, 13) = 11.751, p = 0.004**	F (7, 91) = 0.674, p = 0.694	F (7, 91) = 2.022, p = 0.061
	**LFHS**	**F (1, 13) = 8.005, p = 0.014**	F (7, 91) = 1.605, p = 0.144	F (7, 91) = 1.315, p = 0.252
	**PC**	F (1, 13) = 0.000, p = 0.991	**F (7, 91) = 2.877, p = 0.009**	F (7, 91) = 1.794, p = 0.098
	**HFLS**	F (1, 13) = 0.214, p = 0.652	F (7, 91) = 1.284, p = 0.267	F (7, 91) = 0.671, p = 0.696
	**HFHS**	F (1, 13) = 0.893, p = 0.362	**F (7, 91) = 3.252, p = 0.004**	F (7, 91) = 0.863, p = 0.539
	**Protein**	F (1, 13) = 0.550, p = 0.472	F (7, 91) = 1.142, p = 0.344	F (7, 91) = 0.730, p = 0.647
	**Carb**	F (1, 13) = 0.036, p = 0.530	F (7, 91) = 1.582, p = 0.151	F (7, 91) = 1.168, p = 0.329
	**Sugar**	**F (1, 13) = 11.405, p = 0.005**	F (7, 91) = 0.928, p = 0.489	F (7, 91) = 1.428, p = 0.204
	**Fat**	F (1, 13) = 0.046, p = 0.833	**F (7, 91) = 2.196, p = 0.042**	F (7, 91) = 1.392, p = 0.218
**Bold** = p < 0.05.				

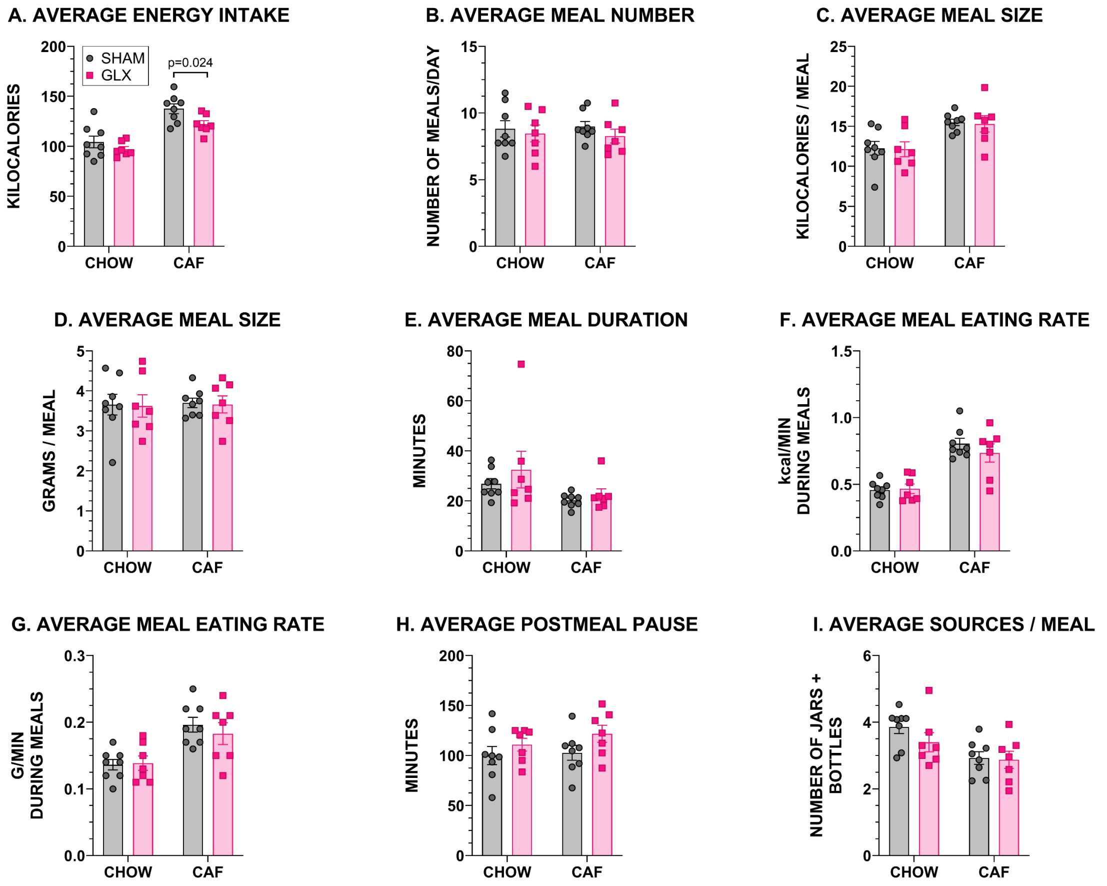

Figure S4 Average energy intake and meal parameters during CHOW and CAF shown as mean±SE (SHAM n=8, GLX n=7). **A.** Energy intake **B.** Meals per day **C.** Meal size in kcal **D.** Meal size in grams **E.** Meal duration **F.** Meal eating rate as kcal/min **G.** meal eating rate as grams/min; **H.** Postmeal pause; and **I.** Intake Sources (food jars + fluid bottles) per meal. Averages on each diet were compared with 2-sample ttest and significant differences are indicated. Details of all statistical outcomes may be found in **Table S3**.

**Table S4**. Comparison of average intakes and the ratio of average CAF/average CHOW between surgical groups (2-sample, 2-tailed t-tests).		
Energy intake	PC	t(9.819) = 1.130, p = 0.285
	CAF	**t(12.368) = 2.565, p = 0.024**
	CAF/PC	t(12.653) = 1.371, p = 0.194
No of Meals	PC	t(12.846) = 0.397, p = 0.698
	CAF	t(11.297) = 1.117, p = 0.287
	CAF/PC	t(11.656) = 0.567, p = 0.581
Meal Size (kcal)	PC	t(12.696) = 0.086, p = 0.932
	CAF	t(7.789) = 0.164, p = 0.874
	CAF/PC	t(12.925) = 0.203, p = 0.842
Meal Size (g)	PC	t(12.696) = 0.086, p = 0.932
	CAF	t(9.490) = 0.160, p = 0.877
	CAF/PC	t(10.790) = 0.283, p = 0.782
Meal Dur	CHOW	t(6.927) = -0.740, p = 0.483
	CAF	t(8.028) = -0.891, p = 0.399
	CAF/PC	t(9.497) = -0.271, p = 0.792
Meal Eating Rate (kcal/min)	PC	t(10.527) = 0.208, p = 0.839
	CAF	t(10.001) = 0.871, p = 0.404
	CAF/PC	t(12.933) = 0.925, p = 0.372
Meal Eating Rate (g/min)	PC	t(10.527) = -0.208, p = 0.839
	CAF	t(10.676) = 0.881, p = 0.398
	CAF/PC	t(12.986) = 0.993, p = 0.339
Post meal Pause	PC	t(12.184) = -0.98, p = 0.346
	CAF	t(12.395) = -1.687, p = 0.117
	CAF/PC	t(9.795) = -0.437, p = 0.672
No Intake Sources	PC	t(10.911) = 1.327, p = 0.212
	CAF	t(11.383) = 0.156, p = 0.879
	CAF/PC	t(11.086) = -1.567, p = 0.145
proportion of total energy intake during CAF	LFLS	**t(7.907) = -3.552, p = 0.008**
	LFHS	t(12.453) = 2.098, p = 0.057
	PC	t(11.819) = 0.745, p = 0.471
	HFLS	t(9.904) = -1.242, p = 0.243
	HFHS	t(9.868) = 1.905, p = 0.086
	PROTIEN	t(9.407) = 0.460, p = 0.656
	FAT	t(11.819) = 0.745, p = 0.471
	CARBOHYDRATE	t(9.278) = -0.532, p = 0.607
	SUGAR	**t(12.976) = 3.928, p = 0.002**
	FAT	t(11.819) = 0.745, p = 0.471
**Bold** = p < 0.05.		

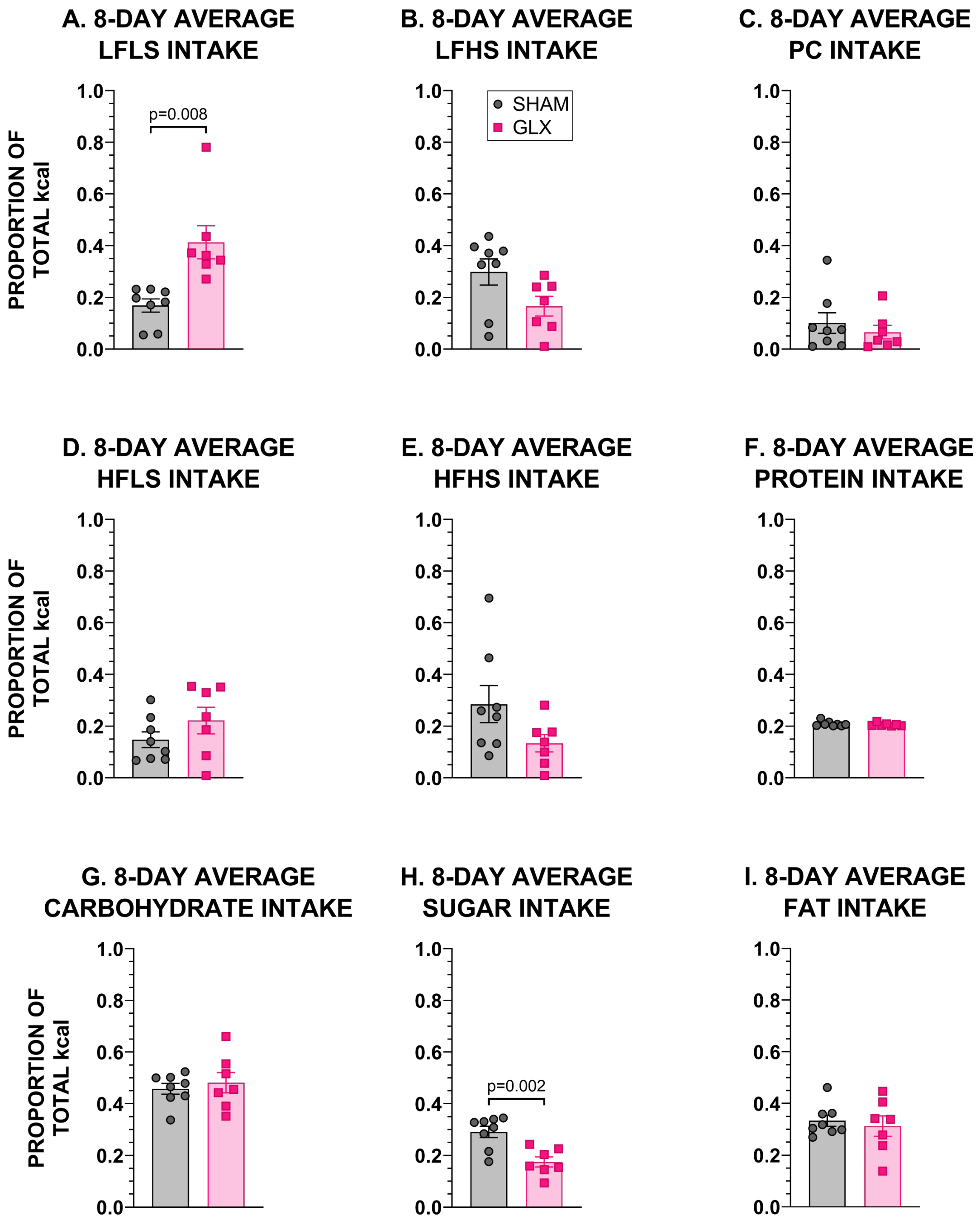

Figure S5. Average intake of individual foods and macronutrients during CAF shown as mean±SE (SHAM n=8, GLX n=7). **A.** LFLS intake **B.** LFHS intake **C.** PC intake **D.** HFLS intake **E.** HFHS intake **F.** protein intake **G**. carbohydrate intake **H**. sugar intake and **I**. fat intake. Averages were compared with 2-sample t-test and significant differences are indicated. Details of all statistical outcomes may be found in **Table S3**.

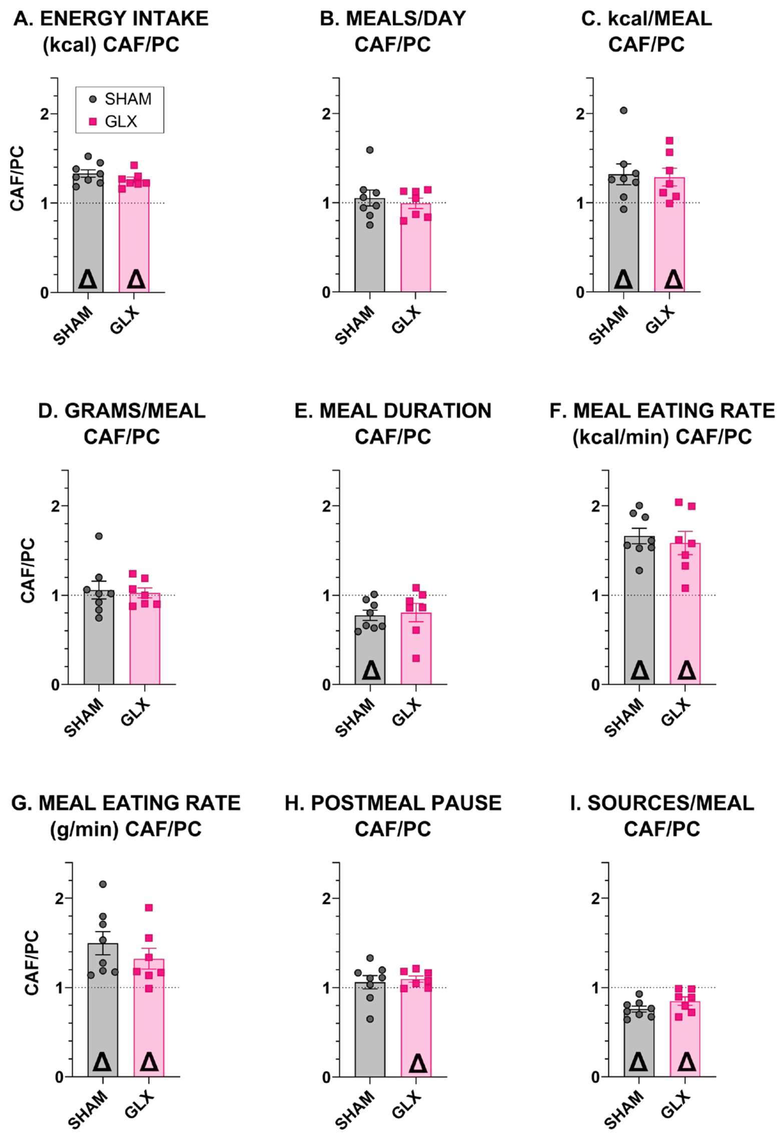

Δ = CAF/CHOW significantly different from 1
(CAF/PC = 1 indicates CAF = PC)

Figure S6. Magnitude of difference in energy intake and meal parameters between CHOW and CAF shown as average on CAF/average on PC. There were no differences between the groups and change scores different from 1 are indicated within the bar for each group by **Δ**. Change from PC of **A**. Energy intake, **B**. Meals per day **C**. Meal size as kcal, **D**. Meal size as grams, **E**. Meal duration, **F**. Meal eating rate as kcal/min, **G**. Meal eating rate as grams/min, **H**. Postmeal pause, and **I**. Intake sources (food jars + fluid bottles) per meal. Details of statistical comparison between groups may be found in **Table S4** and of the magnitude of change of each group in **Table S5**.

**Table S5.** Within-group comparison of average on CAF/average on PC to 1 (1-sample 2-tailed t-test)		
Energy Intake	SHAM	**t(7) = 8.077, p < 0.001**
	GLX	**t(6) = 8.111, p < 0.001**
No of Meals	SHAM	t(7) = 0.6, p = 0.568
	GLX	t(6) = -0.116, p = 0.912
Meal Size (kcal)	SHAM	**t(7) = 2.74, p = 0.029**
	GLX	**t(6) = 2.886, p = 0.028**
Meal Size (g)	SHAM	t(7) = 0.583, p = 0.578
	GLX	t(6) = 0.465, p = 0.658
Meal Dur	SHAM	**t(7) = -3.97, p = 0.005**
	GLX	t(6) = -1.895, p = 0.107
Meal Dur	SHAM	**t(7) = -3.97, p = 0.005**
	GLX	t(6) = -1.895, p = 0.107
Meal Rate (kcal/min)	SHAM	**t(7) = 4.613, p = 0.002**
	GLX	**t(6) = 3.942, p = 0.008**
Post-meal Pause	SHAM	t(7) = 0.820, p = 0.439
	GLX	**t(6) = 2.812, p = 0.031**
Intake Sources /Meal	SHAM	**t(7) = -7.299, p < 0.001**
	GLX	**t(6) = -3.221, p = 0.018**
**Bold =** p < 0.05		
